# *Saccharomyces boulardii* Ameliorates Dextran Sulfate Sodium-Induced Ulcerative Colitis in Mice by Regulating NF-*κ*B and Nrf2 Signaling Pathways

**DOI:** 10.1155/2021/1622375

**Published:** 2021-07-28

**Authors:** Hui Gao, Yinzheng Li, Jie Sun, Huzi Xu, Meng Wang, Xuezhi Zuo, Qiang Fu, Yanchao Guo, Zhenyan Chen, Piwei Zhang, Xudong Li, Niwen Wang, Ting Ye, Ying Yao

**Affiliations:** ^1^Department of Clinical Nutrition, Tongji Hospital, Tongji Medical College, Huazhong University of Science and Technology, Wuhan, Hubei, China; ^2^Department of Nephrology, Tongji Hospital, Tongji Medical College, Huazhong University of Science and Technology, Wuhan, Hubei, China; ^3^Department of Oncology, Tongji Hospital, Tongji Medical College, Huazhong University of Science and Technology, Wuhan, Hubei, China

## Abstract

*Saccharomyces boulardii* (*S. boulardii*) is a probiotic yeast that is widely used to treat gastrointestinal disorders. The present study is aimed to explore the therapeutic effects of *S. boulardii* on dextran sulfate sodium- (DSS-) induced murine ulcerative colitis (UC) and illustrate the mechanisms of action. C57BL/6 mice were administered *S. boulardii* (10^5^ and 10^7^ CFU/ml, *p.o.*) for 3 weeks and then given DSS [2.5% (*w*/*v*)] for one week. Administration of *S. boulardii* prevented DSS-induced reduction in body weight, diarrhea, bloody feces, decreased colon length, and loss of histological structure. Moreover, *S. boulardii* protected the intestinal barrier by increasing the levels of tight junction proteins zona occludens-1 and Occludin and exerted immunomodulatory effects in DSS-induced mice. Furthermore, *S. boulardii* suppressed the colonic inflammation by reducing the levels of Interleukin-1*β*, Interleukin-6, and Tumor necrosis factor alpha and restored myeloperoxidase activity in mice exposed to DSS. *S. boulardii* also mitigated colonic oxidative damage by increasing the levels of antioxidant enzymes (superoxide dismutase, catalase, and heme oxygenase 1) and glutathione and decreasing malondialdehyde accumulation. Further studies identified that *S. boulardii* suppressed the nuclear translocation of nuclear factor kappa B (NF-*κ*B) p65 subunit by decreasing I*κ*K*α*/*β* levels, while promoted the nuclear translocation of nuclear factor erythroid 2-related factor 2 (Nrf2) in DSS-exposed mice. Collectively, *S. boulardii* possessed an appreciable therapeutic effect against the experimental mice model of UC. The protective mechanism of *S. boulardii* may involve inhibition of NF-*κ*B-mediated proinflammatory signaling and activation of Nrf2-modulated antioxidant defense in addition to intestinal barrier protective and immunomodulatory effects.

## 1. Introduction

Ulcerative colitis (UC), one of the inflammatory bowel diseases, is characterized by persistent progression or relapsing inflammation that mainly involves the colonic mucosa and submucosa [1]. The incidence and prevalence of UC have been increasing worldwide in recent years with accelerating circadian rhythm and actuating pressure [2]. The relapse-remission cycles of patients by UC can progress to severe and even face total colectomy and develop colon cancer [3]. Up to now, the specific pathogenesis of UC is still unclear; thus, it is difficult to treat UC in clinic practice. Current treatments (either biological applications or chemical drugs) are not able to obtain a satisfactory curative effect. Severe adverse responses like lymphoma and infection, as well as low efficacy, are always observed [4, 5]. Accordingly, alternative therapies or drugs with low side effects are urgently needed in UC management.

The etiology of UC is complicated, in which genetic susceptibility, hyperreactive immune system, mucosal barrier dysfunction, and alteration of intestinal flora are crucial contributory factors [6, 7]. During the development of colitis, activated immunocytes secrete multiple proinflammatory mediators such as reactive oxygen species (ROS), neutrophil infiltration, and cytokines, which stimulate an inflammatory cascade, resulting in the breakdown of the intestinal epithelial barrier and cell apoptosis, perpetuating chronic intestinal inflammation [8, 9]. Remarkably, nuclear transcription factor kappa B (NF-*κ*B), a key regulatory component in inflammatory process, has been found to be observably activated in UC patients and strongly affects the progression of mucosal inflammation [10]. Moreover, as one of the pivotal transcription factors against inflammation and oxidative stress, nuclear factor erythroid 2-related factor 2 (Nrf2) is reported to be implicated in the suppression of proinflammatory signaling and inflammation-associated pathogenesis [11, 12]. In this regard, it is likely that NF-*κ*B and Nrf2 signaling pathways are promising targets for the molecular therapy of UC.

*Saccharomyces boulardii* (*S. boulardii*) is a probiotic yeast that is resistant to low pH and is highly tolerant to bile acids [13]. It has been received more and more attention in the clinical application in recent years due to its beneficial properties including intestinal immune regulation and intestinal barrier protection [14]. For its few side effects, *S. boulardii* is widely used to treat gastrointestinal disorders, such as traveler's diarrhea, antibiotic-associated diarrhea, tube feeding-associated diarrhea, and acute diarrhea in children [15]. Experimental studies have shown that *S. boulardii* has specific probiotic properties involving impacts on enteric pathogen adhesion, mucosal immune factors, host cell signaling, and mediators of the inflammatory process [15, 16]. *S. boulardii* is able to block NF-*κ*B activation and NF-*κ*B-mediated inflammatory genes expression through producing a low molecular weight soluble factor in intestinal epithelial cells and monocytes [17]. Moreover, a recent study has shown that *S. boulardii* is capable of reducing ROS accumulation via activating Nrf2 pathway, thereby counteracting oxidative stress [18]. However, whether *S. boulardii* has the protective effect on UC and whether this benefit involves modulating oxidative stress and inflammatory reaction are yet poorly known.

Hence, the current study was aimed to examine the therapeutic effect of *S. boulardii* on a well-established mice model of colitis induced by dextran sodium sulfate (DSS) that mimics many clinical symptoms of human UC and, further, investigate the potential mechanisms involved.

## 2. Experimental Section

### 2.1. Chemicals

The lyophilized preparation of *S. boulardii* was obtained from Angel Nutritech Co., Ltd (Hubei, China. 2017081701). Commercial DSS was purchased from MP Biomedicals (Irvine, CA, USA). In addition, other chemicals mentioned in this paper were also laboratory specific.

### 2.2. Animals and Treatment

The animal experiments involved in this article were approved by the Animal Care and Use Committee (ACUC) of Tongji Medical College, Huazhong University of Science and Technology approval (s2135).

**Figure 1:**
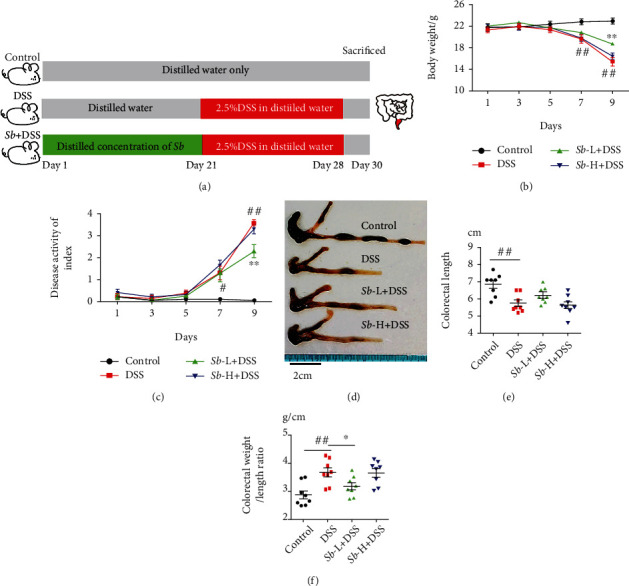
*S. boulardii* alleviated the symptoms in mice with ulcerative colitis. Mice were continuously administrated with 2.5% DSS for 7 days with or without *Sb* (10^5^ and 10^7^ CFU/ml) pretreatment for 21 days. (a) Scheme of the experiment. (b) Weight changes in mice. (c) DAI was recorded every other day. (d) The representative pictures of colon. (e) Average colorectal lengths. (f) Colorectal weight/length ratio in each group. *N* = 8. Values are presented as mean ± SEM. ^#^*P* < 0.05 and ^##^*P* < 0.01 compared with the control group. ^∗^*P* < 0.05, and ^∗∗^*P* < 0.01 compared with the other groups. *Sb*: *S. boulardii*.

Female C57BL/6 mice (8 weeks old, 20-24 g) were from Beijing HuaFuKang Laboratory Animal Technology Co., Ltd. (Beijing, China). Animals were kept in a specific pathogen-free (SPF) environment with suitable temperature (25 ± 2°C) and humidity (55%), and the room followed a 12/12-hour light and dark cycle. These mice were provided with adequate amounts of food and water. Every fifth mouse was placed in a plastic cage. After one week of adaptive feeding, the mice were randomly divided into four groups, namely, the control group (noncolitis), the DSS group (colitis), the *Sb* − L group (10^5^ CFU/ml) + DSS group, and the *Sb* − H group (10^7^ CFU/ml) + DSS group (*N* = 20 per group). Mice were treated with distilled water or *S. boulardii* suspension by oral gavage for 21 consecutive days. On the 22nd day, the mice received 2.5% (wt/vol) DSS for 7 days to induce ulcerative colitis (Figure 1(a)). The doses of suspension were allocated by 10^5^ and 10^7^ CFU *S. boulardii* in 300 *μ*L double-distilled water per day, which were proximately 0.1 and 10 folds of clinical doses. The weight changes, gross blood in the feces, and stool consistency were monitored daily for each mouse in a blind manner.

Once the mice were sacrificed, the blood was collected. The colon was removed aseptically and weighed and measured its length as well. The colonic tissue and fecal samples were frozen at -80°C for future use.

### 2.3. Disease Activity Index (DAI)

To assess Disease Activity Index (DAI), the changes in the weight and feces of the mice were observed and recorded every other day. To quantify the score, the Cooper method was slightly modified [19]. The DAI assesses the severity of colitis in mice from three aspects: body weight loss, diarrhea, and blood (Table S1). Weight loss is defined as the difference between the initial weight at the beginning of the experiment and a given day. The four groups were scored for the DAI from the 21^st^ to the 30^th^ day.

### 2.4. Histologic Analysis

After obtaining the mice's distal colon tissues (1-2 cm from the anal verge), placed them in 4% paraformaldehyde solution and fixed them on a shaker for 24 hours. Then, the tissues were dehydrated, embedded, and sliced. To observe the histopathological changes between different groups, the colon tissues were stained with hematoxylin and eosin (HE) and observed and photographed under a microscope (Olympus, Japan).

Colonic histological damage was scored based on the combination of the severity of inflammation, mucosal and crypt damage, and ulceration [20]. The rules of scoring were shown detailly in Table S2. Six random fields were taken from each colon.

### 2.5. Biochemical Determinations in Colonic Tissue

Colonic tissues were lavaged, weighed, and put into normal saline. After tissue samples were homogenized, centrifuge at 4000 r/min for 10 min. The supernatant was collected for further detection of superoxide dismutase (SOD), catalase (CAT), glutathione (GSH), and malondialdehyde (MDA) (Beyotime Biotechnology, Nantong, China). Kits for myeloperoxidase (MPO) analysis were from Jiancheng Bioengineering Institute (Nanjing, China). The detection is carried out according to the instructions of the commercial kits.

### 2.6. Terminal Deoxynucleotidyl Transferase Mediated dUTP Nick-End Labeling (TUNEL) Assay

To assess apoptosis in colonocytes, the fluorescein (FITC) Tunel Cell Apoptosis Detection Kit (Servicebio, China) was used to perform TUNEL assay. Specifically, the paraffin-embedded colons were cut into 4 *μ*m thick sample sections. After the sample sections were deparaffinized and rehydrated, incubated them with TUNEL reaction mixture in a 37°C incubator for 60 min. After that, incubated the samples with 4,6-diamidino-2-phenylindole (DAPI) (Roche, Switzerland) for 10 min. The results of TUNEL assay could be observed under a microscope (Olympus, Japan). Cells that were positive for apoptosis showed bright green nuclear staining. In each sample, six randomly optical areas were taken to quantify the staining.

### 2.7. Immunofluorescence Staining

The tissue sample sections were heated in EDTA/citrate antigen retrieval solution. The sections were deparaffinized with xylene, hydrated with ethanol of different concentration gradients; blocked with serum at room temperature for 30 minutes; then incubated with specific primary antibodies such as ZO-1 (1 : 100), Occludin (1 : 100), F4/80 (1 : 100), Ly6G (1 : 100), NF-*κ*B p65 (1 : 100), and Nrf2 (1 : 100) overnight at 4°C; and followed by fluorescently labeled secondary antibodies in 37°C incubator for 60 min. After counterstaining with DAPI, the colon samples were observed and photographed by a microscope (Olympus, Japan). In each sample, six randomly optical areas were taken to analyze.

### 2.8. RNA Preparation and Q-PCR

Trizol reagent (Invitrogen, USA) was used to extract total RNA from colon tissues according to the instructions of the manufacturer. Single-stranded cDNA was reverse transcribed from RNA by a reverse transcription kit (Takara, Japan). Quantitative PCR was performed on the Roche light 480II. The mRNA levels of Interleukin-1*β* (*Il1b*), Interleukin-6 (*Il6*), Tumor necrosis factor alpha (*Tnf*), Transforming growth factor-*β*1 (*Tgfb1*), Tight junction proteins zona occludens-1 (*Tjp1*), and Occludin (*Ocln*) were measured by Q-PCR. The mRNA relative expression levels were normalized to the expressions of *Gapdh*. The primers used are listed in Table 1.

### 2.9. Western Blot

Colon tissues were homogenized in the protein lysis mix. The same amount of proteins was loaded on sodium dodecylsulfatepolyacrylamide gel and transferred to PVDF membranes (Millipore, USA). After blocking with skim milk for 60 minutes at room temperature, the PVDF membranes were incubated with corresponding primary antibodies at 4°C overnight. The following primary antibodies were used: rabbit anti-Occludin (1 : 1000, Abcam, UK), rabbit anti-ZO-1, anti-Nrf2, anti-heme oxygenase 1 (HO-1) (1 : 1000, ABclonal, China), rabbit anti-NF-*κ*B p65, anti-I*κ*K*α*, anti-I*κ*K*β*, anti-Phospho-NF-*κ*B p65 (Ser536) (1 : 1000, Cell Signaling Technology, USA), and mouse anti-GAPDH (1 : 5000, Abbkine, China). HRP-conjugated secondary antibodies (1 : 4000, ABclonal, China) were used to incubate the blots, and the blots were observed by enhanced chemiluminescence (Bio-Rad, USA). The relative intensity of the target blot was quantified by Image J (NIH, USA). Four independent experiments were performed.

### 2.10. Flow Cytometry and Cell Sorting

The colon was digested into a single-cell suspension. Cells were incubated with PE/Cy7-conjugated anti-mouse CD45 (eBoscience, USA), FITC-conjugated anti-mouse CD3, BV421-conjugated anti-mouse CD4, PE-conjugated anti-mouse CD8, APC-conjugated anti-mouse/human CD11b, and PE-conjugated anti-mouse F4/80 (BioLegend, USA) at room temperature in dark for 30 min for fluorescence-activated cell sorting (FACS) flow cytometry.

### 2.11. Statistical Analysis

All results are showed as mean ± SEM, and the differences among the means were determined by one-way analysis of variance (ANOVA) using SPSS 21.0 (IBM SPSS, Inc., USA). *P* < 0.05 was considered statistical significance.

## 3. Results

### 3.1. *S. boulardii* Alleviates the Clinical Symptoms of DSS-Induced Colitis

Mice exposed to DSS showed significant weight loss from the seventh day (Figure 1(b)), and correspondingly, a notable increase in DAI (Figure 1(c)) evidenced by apparent diarrhea and rectal bleeding when compared with the control animals. Moreover, DSS-treated mice displayed a significant reduction in colon length (Figures 1(d) and 1(e)), while a marked increase in colon weight/length ratio (Figure 1(f)) in comparison to controls. However, administration of *S. boulardii* at low dosage significantly prevented the weight loss, improved the DAI, and decreased the colon weight/length ratio in DSS-exposed mice (Figures 1(b)–1(f)). Collectively, these results suggest that *S. boulardii* alleviates the clinical symptoms of colitis in DSS-exposed mice.

### 3.2. *S. boulardii* Attenuates Histopathological Changes in DSS-Induced Colitis

**Figure 2:**
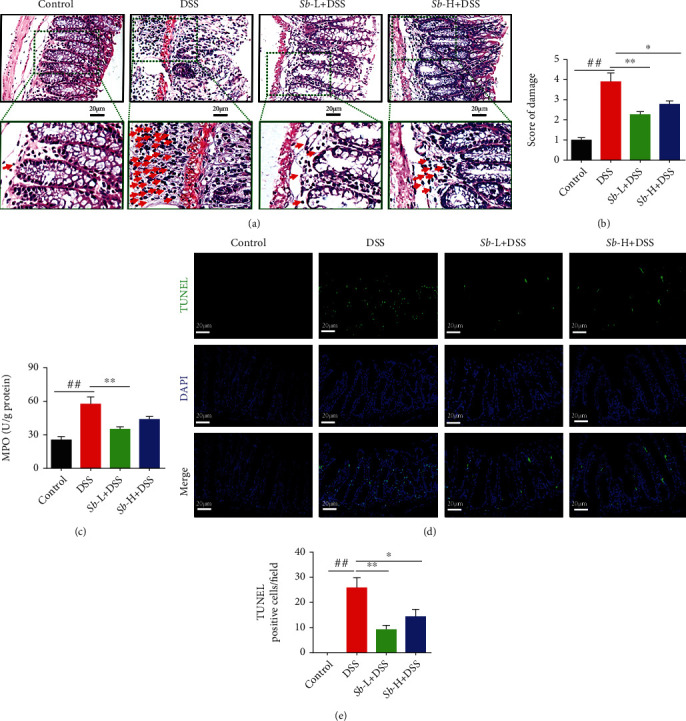
*S. boulardii* alleviated pathological alterations in ulcerative colitis. (a) Representative images of colon injury shown by hematoxylin and eosin (HE) staining (Magnification: ×400; Scale bars: 20 *μ*m). Arrows indicated the infiltration of neutrophils and monocytes in mucosa. (b) Histopathological score on colons. (c) Colonic MPO level. (d) Representative colon images of colon cell apoptosis. (e) Quantification of TUNEL positive cell number/field. *N* = 8. Values are presented as mean ± SEM. ^#^*P* < 0.05 and ^##^*P* < 0.01 compared with the control group. ^∗^*P* < 0.05, and ^∗∗^*P* < 0.01 compared with the other groups. *Sb*: *S. boulardii*.

Colon tissues from control mice were free of histological lesions. In contrast, mice with ulcerative colitis exhibited severe damage in intestinal segments, including the loss of histological architecture, decreased in crypt numbers, and infiltration of granulocytes and monocytes in the mucosa and submucosa (Figure 2(a)). Additionally, the severity of DSS-induced colitis was evaluated, and the histopathological damage score was higher in DSS-exposed mice than in control animals (Figure 2(b)). However, *S. boulardii* effectively alleviated these histological lesions and decreased the damage score in DSS-treated mice (Figures 2(a) and 2(b)).

To further confirm the degree of neutrophil infiltration, MPO activity, which positively reflects the number of neutrophil granulocytes, was determined. As shown in Figure 2(c), MPO activity was markedly increased in DSS-treated mice versus the normal controls (*P* < 0.01), which was significantly alleviated by administration of *S. boulardii* (10^5^ CFU/ml) (*P* < 0.01).

To investigate the role of apoptosis in DSS-induced colitis and the antiapoptotic effect of *S. boulardii*, TUNEL assay was performed. As depicted in Figure 2(d), the colonic epithelial cells of control animals were rarely TUNEL positive, while DSS-treated mice showed a dramatic elevation in TUNEL-positive cells (*P* < 0.01). Nevertheless, treatment with *S. boulardii* (10^5^ and 10^7^ CFU/ml) significantly prevented the increase in colonic epithelial cell apoptosis in DSS-exposed mice (Figure 2(e)).

### 3.3. *S. boulardii* Protects against Intestinal Barrier Dysfunction in DSS-Induced Colitis

**Figure 3:**
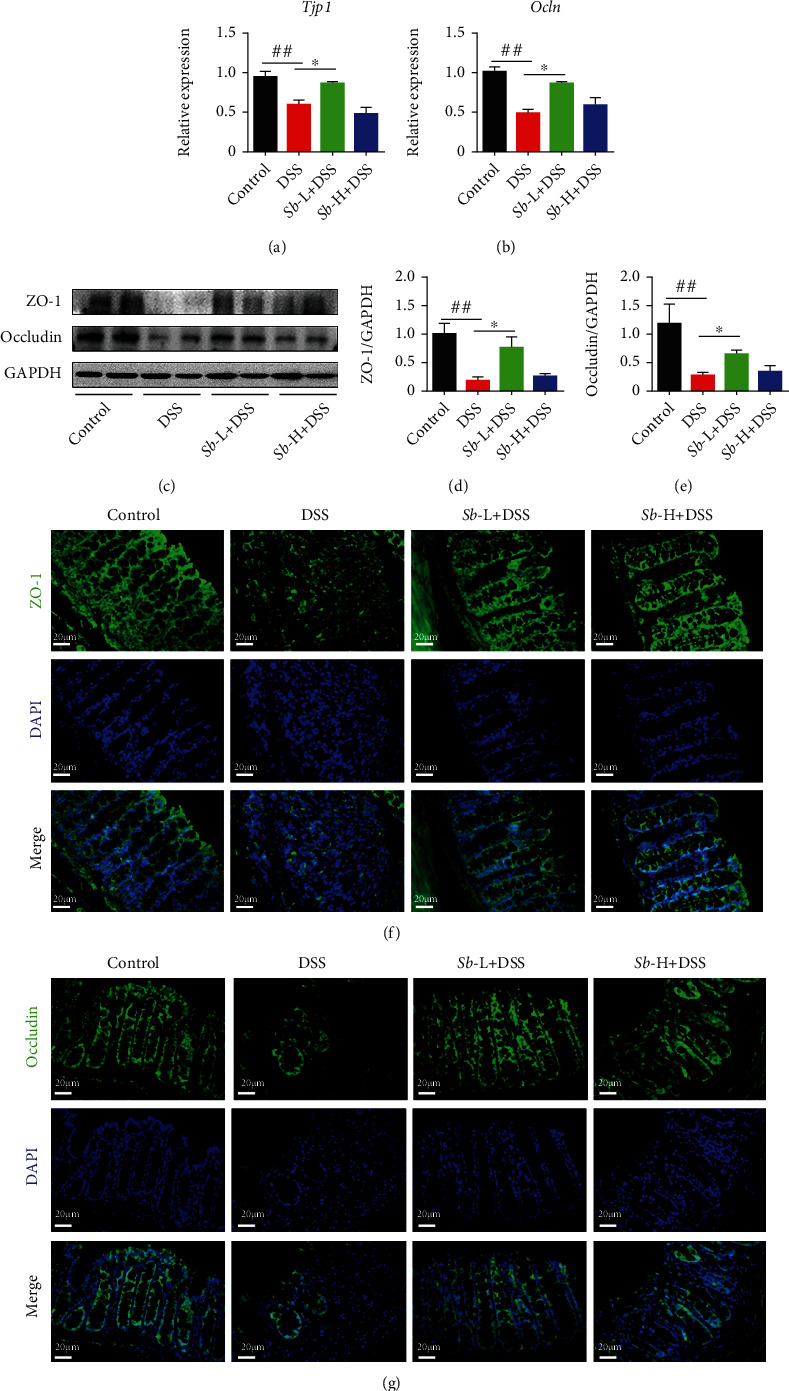
*S. boulardii* alleviated pathological alterations in colitis induced by DSS. Relative expression of mRNA in the colon (a) *Tjp1* and (b) *Ocln*. Representative western blot pictures (c) and quantification of (d) ZO-1 and (e) Occludin. (f, g) Representative immunofluorescence images of ZO-1 (f, green), Occludin (g, green) in distal ileum (Magnification: ×400; Scale bar: 20 *μ*m). DAPI was used for nuclear counterstaining (blue). *N* = 8. Values are presented as mean ± SEM. ^##^*P* < 0.01 compared with the control group. ^∗^*P* < 0.05 versus the other groups. *Sb*: *S. boulardii*.

To explore the effect of *S. boulardii* on intestinal barrier dysfunction in mice exposed to DSS, the levels of ZO-1 and Occludin, two key proteins within tight junctions and essential to the integrity of the epithelial and endothelial barriers, were determined. As shown in Figures 3(a) and 3(b), the mRNA levels of *Tjp1* and *Ocln* were significantly reduced in DSS-exposed mice as compared with the control animals. However, treatment with low-dosage *S. boulardii* uniformly elevated *Tjp1* and *Ocln* levels in DSS-exposed mice (both *P* < 0.05). At the same time, the protein levels of ZO-1 and Occludin were obviously decreased in the colons from DSS-exposed mice as compared to the controls (both *P* < 0.01, Figures 3(c)–3(e)). Nevertheless, administration of *S. boulardii* (10^5^ CFU/ml) significantly suppressed these alterations. Consistently, immunofluorescence of tight junctions in the distal ileum demonstrated that *S. boulardii* administration ameliorated the changes in ZO-1 and Occludin levels induced by DSS (Figures 3(f) and 3(g)). These results suggest that *S. boulardii* attenuates the DSS-induced intestinal barrier dysfunction through promoting ZO-1 and Occludin expression.

### 3.4. *S. boulardii* Displays Immunomodulatory Effects in DSS-Induced Colitis

**Figure 4:**
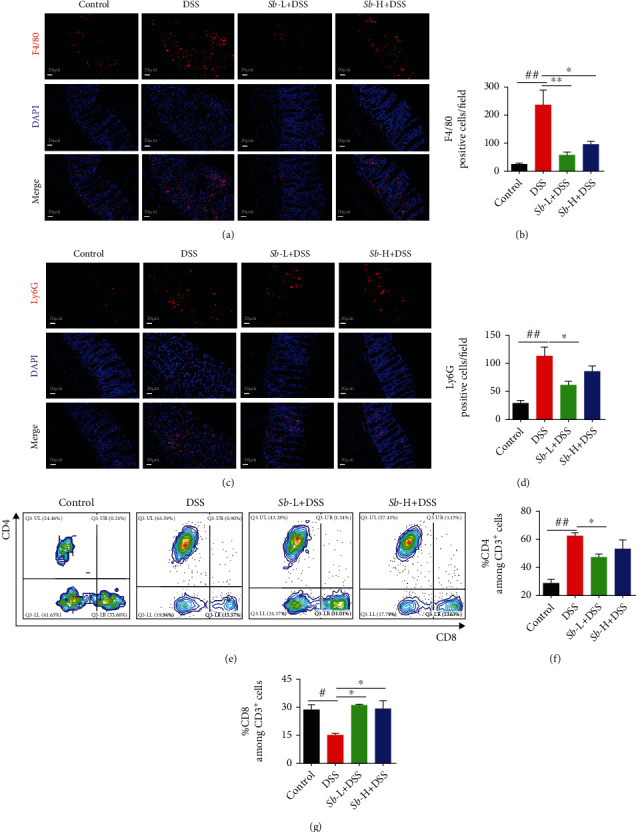
The immunomodulatory effects of *Sb* (10^5^ and 10^7^ CFU/ml) on colons of DSS-induced colitis mice. (a) Immunofluorescence staining analysis of F4/80-positive macrophages (red) in colon mucosa. (Magnification: ×200; Scale bar: 20 *μ*m). DAPI was used for nuclear counterstaining (blue). (b) Statistics of F4/80. *N* = 8. (c) Immunofluorescence analysis of Ly6G positive neutrophils (red) in colon mucosa. (Magnification: ×200; Scale bar: 20 *μ*m). DAPI was used for nuclear counterstaining (blue). (d) Statistics of Ly6G. *N* = 8. (e–g) The CD4^+^ and CD8^+^ T cell percentages were detected by flow cytometry. *N* = 4. Values are presented as mean ± SEM. ^#^*P* < 0.05 and ^##^*P* < 0.01 compared with control group. ^∗^*P* < 0.05, and ^∗∗^*P* < 0.01 compared with the other groups. *Sb*: *S. boulardii*.

Next, the immunomodulatory properties of *S. boulardii* were characterized in colitis mice, involving in macrophages, neutrophils, and the intestinal immune response. First, the level of the F4/80, a unique marker of murine macrophage, was assessed. As exhibited in Figures 4(a) and 4(b), the intestinal macrophages expressing F4/80 were notably accumulated in DSS-exposed mice compared to those in controls (*P* < 0.01). Interestingly, administration of *S. boulardii* at both concentrations (10^5^ and 10^7^ CFU/ml) resulted in a significant reduction of F4/80^+^ macrophages in DSS-treated mice (both *P* < 0.05).

And then, the neutrophil surface antigen Ly6G, which regulates leukocyte migration and neutrophil recruitment, was examined. As exhibited in Figures 4(c) and 4(d), an obvious neutrophil infiltration in colon evidenced by Ly6G staining was seen in DSS-treated mice when compared to control animals (Figures 4(c) and 4(d)). Of note, the inhibition of Ly6G^+^ granulocytes recruitment was achieved only by 10^5^ CFU/ml *S. boulardii* treatment (*P* < 0.05).

Finally, the percentages of CD4^+^ and CD8^+^ T cells, which are very important for immune defense, were determined. As presented in Figures 4(e)–4(g), the percentage of CD4^+^ T cells was dramatically increased, while CD8^+^ T cells were obviously decreased in DSS-exposed mice compared with the controls (both *P* < 0.05). However, *S. boulardii* treatment at both dosages (10^5^ and 10^7^ CFU/ml) reversed these changes in DSS-treated mice (both *P* < 0.05). These data indicate that the administration of *S. boulardii* regulates the immune response in colitis mice.

### 3.5. *S. boulardii* Modulates Inflammation by Suppressing NF-*κ*B Signaling in Colitis Mice

**Figure 5:**
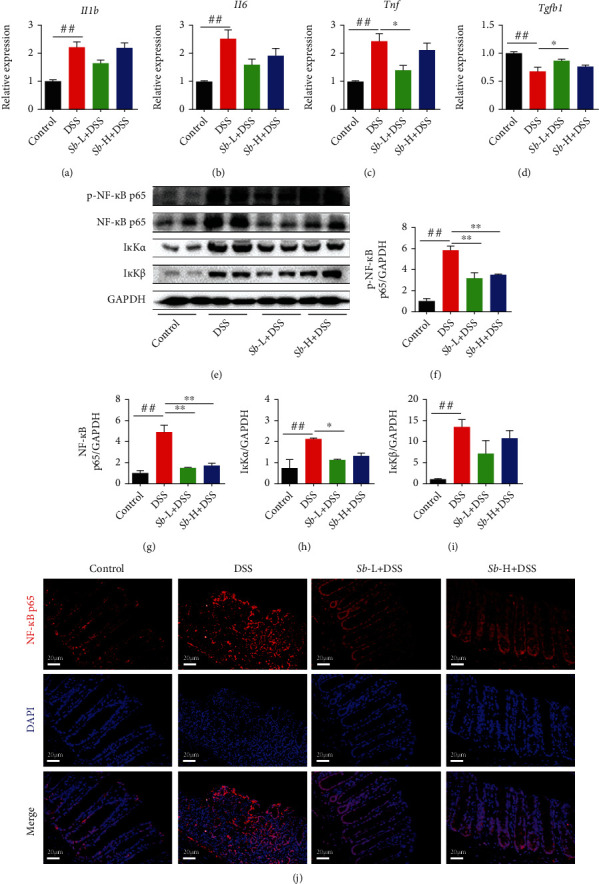
Inhibitory effect of *S. boulardii* on NF-*κ*B signaling pathway in ulcerative colitis induced by DSS in mice. Relative expression of mRNA in the colon (a) *Il1b*, (b) *Il6*, (c) *Tnf*, and (d) *Tgfb1*. Representative western blot images (e) and quantification of (f) p-NF-*κ*B p65, (g) NF-*κ*B p65, (h) I*κ*K*α*, and (i) I*κ*K*β*. (j) Immunofluorescence analysis of NF-*κ*B p65 (red) in colon (Magnification: ×400; Scale bars: 20 *μ*m). DAPI was used for nuclear counterstaining (blue). *N* = 6. Values are presented as mean ± SEM. ^#^*P* < 0.05 versus control group. ^∗^*P* < 0.05, and ^∗∗^*P* < 0.01 compared with the other groups. *Sb*: *S. boulardii*.

To identify further the inflammation modulatory effect of *S. boulardii* on DSS-elicited colitis, the levels of several inflammatory cytokines mRNA (*Il1b*, *Il6*, *Tnf*, and *Tgfb1*) were measured by Q-PCR. As depicted in Figures 5(a)–5(d), the levels of *Il1b*, *Il6*, and *Tnf* were significantly increased in the colon of DSS-exposed mice in comparison with the control animals (all *P* < 0.01). However, administration of *S. boulardii*, especially at the low dosage, suppressed the upregulation of those cytokines in the colons of mice exposed to DSS (all *P* < 0.05). In addition, *Tgfb1* level was profoundly decreased in the inflamed colonic tissue of DSS-exposed mice while reversed by *S. boulardii* treatment (*P* < 0.05).

Since the NF-*κ*B signaling pathway has a vital role in the inflammatory process, the key components of this pathway were assessed by western blot. As compared to the controls, the levels of p-NF-*κ*B p65, NF-*κ*B p65, and inhibitor of kappa B (I*κ*B) kinase *α*/*β* (I*κ*K*α*/*β*) were obviously increased in the colons from DSS-exposed mice (all *P* < 0.01, Figures 5(e)–5(i)), indicative of NF-*κ*B signaling activation. Nevertheless, administration of *S. boulardii* (10^5^ and 10^7^ CFU/ml) significantly suppressed these alterations. Of note, the degradation of I*κ*K*α* but not I*κ*K*β* was only observed in mice treated with 10^5^ CFU/ml *S. boulardii* (*P* < 0.05).

To further confirm NF-*κ*B activation, the subcellular localization of the active subunit NF-*κ*B p65 was evaluated. Immunofluorescence results demonstrated that the NF-*κ*B p65 subunit was low intensity and mainly located in the cytoplasm in the control colons. Nonetheless, it has a much higher intensity than in the controls and is mainly located in the nucleus in the colons of DSS-exposed mice. As expected, administration of *S. boulardii* (10^5^ and 10^7^ CFU/ml) markedly reduced NF-*κ*B p65 staining in the colons of DSS-exposed mice (Figure 5(j)). Taken together, these findings suggest that *S. boulardii* modulates inflammation by suppressing NF-*κ*B signaling in colitis mice.

### 3.6. *S. boulardii* Activates Nrf2 Signaling to Reduce Oxidative Stress in Colitis Mice

**Figure 6:**
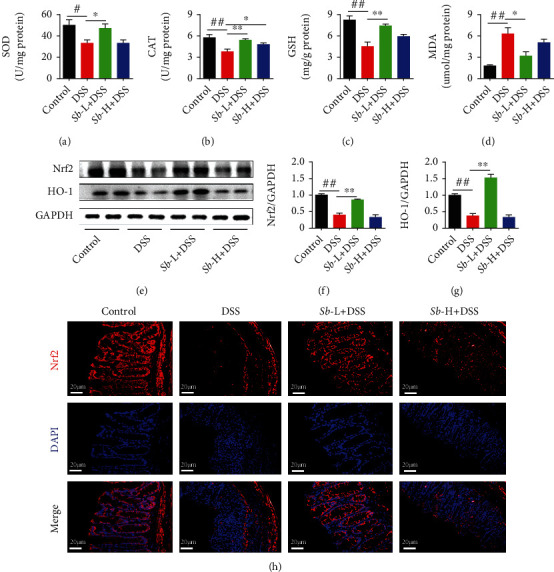
Detraction effect of *S. boulardii* on Nrf2 signaling pathway in DSS-induced colitis mice. Colon oxidative stress-related indicators (a) SOD, (b) CAT, (c) GSH, and (d) MDA. Representative western blot images (e) and quantification of (f) Nrf2 and (g) HO-1. (h) Immunofluorescence staining of Nrf2 (red) in colon (magnification: ×400; scale bar: 20 *μ*m). DAPI was used for nuclear counterstaining (blue). *N* = 6. Values are presented as mean ± SEM. ^#^*P* < 0.05 and ^##^*P* < 0.01 compared with control group; ^∗^*P* < 0.05 and ^∗∗^*P* < 0.01 compared with the DSS groups. *Sb*: *S. boulardii*.

In inflammatory diseases such as UC, inflammatory cells and epithelial cells generated a large amount of ROS, causing direct damage to colon epithelial cells and contributing to UC development [21, 22]. To test the antioxidant capacity of *S. Boulardii* in UC, the levels of oxidative stress markers were examined. As showed in Figure 6, the levels of SOD, CAT, and GSH in colons of mice exposed to DSS were notably reduced, while the level of MDA was obviously increased when compared to the control animals (all *P* < 0.05), suggestive of oxidative stress induced by DSS. However, treatment with *S. Boulardii* significantly relieved the oxidative stress by enhancing colonic SOD, CAT, and GSH levels and reducing colonic MDA level in DSS-exposed mice (all *P* < 0.05).

Since antioxidant enzyme gene expression is regulated by Nrf2 signaling pathway, the underlying mechanism implicated in the antioxidative effect of *S. boulardii* on DSS-induced colitis was explored by evaluating the expression and distribution of Nrf2 proteins. As shown in Figures 6(e)–6(g), the level of Nrf2 and HO-1 was significantly reduced in mice exposed to DSS compare to the controls (*P* < 0.01). However, *S. boulardii* significantly enhanced its level in DSS-treated mice (*P* < 0.01). Moreover, the effect of *S. boulardii* on Nrf2 activation was confirmed by immunofluorescence analysis of Nrf2 in colon mucosa cells (Figure 5(h)). In addition to activating the transcription of various antioxidant enzyme genes, Nrf2 is also able to activate the genes of transcription of phase II detoxification enzyme such as HO-1. As expected, *S. boulardii* notably reversed the DSS-induced decrease in HO-1 level in colons of treated mice. Collectively, these results suggest that *S. boulardii* mitigates oxidative stress by activating Nrf2 signaling in colitis mice.

## 4. Discussion

In this study, mice exposed to DSS demonstrated an elevated DAI with body weight loss, diarrhea, bloody feces, and decreased colon length. These were accompanied by severe histological alterations including the loss of morphological structure, reduction in number of crypts, and inflammatory cell infiltration. Those clinical signs resemble and are comparable to the symptoms usually observed in human UC [23, 24], suggestive of successful establishment of DSS-induced colitis mice model in this research. The present findings are consistent with previous studies [24–26]. Moreover, DSS induced excessive cell apoptosis in colonic tissue, which may play a crucial role in these observed morphological alterations. Additionally, DSS exposure displayed epithelial barrier dysfunction, evidenced by the decreased levels of tight junction proteins ZO-1 and Occludin in colons of mice. However, administration of *S. boulardii* reestablished the gut health and exerted a significant effect on structural and functional maintenance of colons in DSS-treated mice, demonstrating the promising preventive potential in UC.

Although the underlying specific etiologic causes and mechanisms of UC are not completely clear yet, the immune response abnormality has attracted more and more attention [27]. In the present study, DSS exposure induced a significant elevation in F4/80^+^ macrophages, Ly6G^+^ granulocytes, and CD4^+^ T cells in mice colons, indicating immune response disorder. However, administration of *S. boulardii* exerted its immunomodulatory effects by reversing these alterations in DSS-treated mice. In addition, inflammatory response has also been implicated in the pathogenesis of UC [1]. Increasing evidence has shown that the proinflammatory cytokines result in intestinal tissue damage by amplifying the inflammatory cascade in DSS-induced UC [28, 29]. In agreement with this, the current work demonstrated a notable increase in the levels of *Il1b*, *Il6*, and *Tnf* in mice exposed to DSS. These observations are in line with previous findings from patients with UC [30] and in experimental colitis [31]. The levels of proinflammatory cytokines are affected by inflammatory cells including neutrophils, macrophages, and leukocytes. Infiltration of inflammatory cells in the colon has been suggested to contribute to the mucosal tissue impairment, mucosal barrier disruption, and subsequent inflammatory response [32]. This is further supported by the present result on elevated MPO activity, which is an index of colon mucosa neutrophil infiltration, a marker of acute inflammation [33]. Interestingly, administration of *S. boulardii* greatly reduced the activity of MPO and inhibited the expression of those cytokines in DSS-induced colitis mice, indicating that the protective effect of *S. boulardii* against colonic damage is associated with the regulation of inflammatory cytokines. Together, these observations indicate the ability of *S. boulardii* to modulate immune response and prevent inflammatory cells infiltration and inflammatory response in the colonic tissues of DSS-exposed mice.

The present study also provided insights into the molecular mechanism through which *S. boulardii* exerted its anti-inflammatory effect in DSS-induced colitis. It is known that the transcription of inflammatory cytokines relies on NF-*κ*B activation, which is triggered by a sequential cascade including degradation of its inhibitory subunit I*κ*B*α* by I*κ*B kinases I*κ*K*α*/*β*, as well as nuclear translocation of the cytosolic p65 subunit of NF-*κ*B [34]. The current work showed that the levels of p-NF-*κ*B p65 and I*κ*K*α*/*β* in colons of DSS-exposed mice were significantly increased compared with control animals. Moreover, an intensive staining of NF-*κ*B p65 subunit in nucleus was also observed in mice exposed to DSS, further confirming that the NF-*κ*B signaling pathway was activated by DSS. Nonetheless, administration of *S. boulardii* downregulated the levels of p65 NF-*κ*B, I*κ*K*α*, and I*κ*K*β*, as well as the downstream inflammatory cytokines. Hence, it is likely that *S. boulardii* prevented I*κ*B*α* degradation by decreasing I*κ*K*α*/*β* levels, suppressing nuclear translocation of NF-*κ*B, therefore inhibiting activation of NF-*κ*B signaling, finally mitigating DSS-induced colitis. An earlier study by Sougioultzis et al. demonstrated that *S. boulardii* inhibits NF-*κ*B activation and related proinflammatory signaling in host cells by producing a soluble anti-inflammatory factor [17], supporting the present findings. It is worth noting that *S. boulardii* increased *Tgfb1* mRNA expression in the current study. Since TGF-*β*1 induction has been shown to be effective for the recovery of inflammation-related colitis [35], further studies are needed to clarify the regulatory mechanism of inflammatory response by *S. boulardii* when treating colitis and other inflammatory diseases.

It has been shown that inflammation and oxidative stress create a vicious cycle contributing to the pathogenesis of many inflammatory diseases including UC [36]. During colitis, proinflammatory factors activate phagocytes, which are recruited to the mucosa and result in ROS generation [9], causing oxidative stress. Consistent with this, in the present work, DSS exposure induced accumulation of MDA level in the colonic tissues, suggestive of lipid peroxidation and increased production of ROS. The antioxidant defense system comprises the enzymatic and nonenzymatic antioxidants such as SOD, CAT, and GSH, which are responsible for the removal of free radicals and counteracting oxidative stress. The significant reduction in colonic SOD, CAT, and GSH levels was parallel with the marked elevation in MDA level in DSS-exposed mice in the current investigation. These findings suggest the inactivation of antioxidant defense system in the colonic tissues of DSS-exposed mice. Importantly, administration of *S. boulardii* notably improved the DSS-induced reduction in these antioxidants and accumulation of MDA in the exposed mice. Therefore, these observations imply that *S. boulardii* restored colitis-linked depletion of antioxidant system via, at least in part, boosting the levels of enzymatic and nonenzymatic antioxidants in the colons of DSS-exposed mice.

The Nrf2 signaling pathway plays a crucial part in cellular antioxidant defense [37–39]. Under physiological conditions, cytoplasmic protein chaperone Keap1 interacts with Nrf2 to retain the quiescent state. Upon oxidative stress, Nrf2 is able to escape from Keap1 and translocate to the nucleus, where it induces the transcription of a series of genes for antioxidant enzymes (including SOD, CAT, and GSH) as well as genes for phase II detoxification enzymes (such as HO-1) [40]. In the present study, *S. boulardii* significantly increased Nrf2 level and its nuclear localization in colons of DSS-exposed mice. Moreover, these were accompanied by restoration of DSS-induced decline in levels of SOD, CAT, GSH, and HO-1, as well as accumulation of MDA. Taken together, the above results indicate that *S. boulardii* promotes Nrf2 signaling activation to decrease the DSS-induced oxidative injury in colons.

## 5. Conclusion

**Figure 7:**
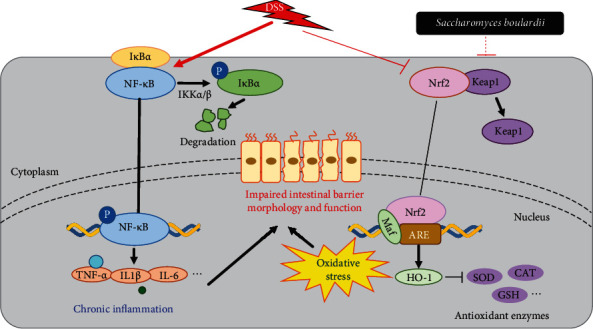
A proposed model for the mechanism of *S. boulardii* against DSS-induced UC.

The current results indicate that the administration of *S. boulardii* is effective for the prevention of DSS-induced mice UC. More importantly, *S. boulardii* exerts attractive intestinal barrier protective and immunomodulatory effects and, particularly, protects against UC via inhibition of NF-*κ*B-mediated proinflammatory signaling and activation of Nrf2-modulated cytoprotective antioxidant defense mechanism (Figure 7). Thus, *S. boulardii* might be a new and attractive therapeutic option for UC.

## Figures and Tables

**Table 1:** Sequences of primers used for Q-PCR.

Gene		Gene sequence
*Il1b*	Forward primer	GTGGCTGTGGAGAAGCTGTG
	Reverse primer	GAAGGTCCACGGGAAAGACAC

*Il6*	Forward primer	GAGGATACCACTCCCAACAGACC
	Reverse primer	AAGTGCATCATCGTTGTTCATACA

*Tnf*	Forward primer	CTGAACTTCGGGGTGATCGG
	Reverse primer	GGCTTGTCACTCGAATTTTGAGA

*Tgfb1*	Forward primer	CAACAATTCCTGGCGTTACCTT
	Reverse primer	TCGAAAGCCCTGTATTCCGTCT

*Tjp1*	Forward primer	GAGCGGGCTACCTTACTGAAC
	Reverse primer	GTCATCTCTTTCCGAGGCATTAG

*Ocln*	Forward primer	TGAAAGTCCACCTCCTTACAGA
	Reverse primer	CCGGATAAAAAGAGTACGCTGG

*Gapdh*	Forward primer	TGACCTCAACTACATGGTCTACA
	Reverse primer	CTTCCCATTCTCGGCCTTG

## Data Availability

The data that support the findings of this study are available from the corresponding author upon reasonable request.

## Supplementary Materials

Supplementary Materials Tables S1-S2 were listed in the Supplementary Material.

## References

[1] Xavier R. J., Podolsky D. K. (2007). Unravelling the pathogenesis of inflammatory bowel disease. *Nature*.

[2] Ananthakrishnan A. N., Kaplan G. G., Ng S. C. (2020). Changing global epidemiology of inflammatory bowel diseases: sustaining health care delivery into the 21st century. *Clinical Gastroenterology and Hepatology*.

[3] Colombel J. F., Narula N., Peyrin-Biroulet L. (2017). Management strategies to improve outcomes of patients with inflammatory bowel diseases. *Gastroenterology*.

[4] Park S. C., Jeen Y. T. (2018). Anti-integrin therapy for inflammatory bowel disease. *World Journal of Gastroenterology*.

[5] Curro D., Pugliese D., Armuzzi A. (2017). Frontiers in drug research and development for inflammatory bowel disease. *Frontiers in Pharmacology*.

[6] Ungaro R., Mehandru S., Allen P. B., Peyrin-Biroulet L., Colombel J. F. (2017). Ulcerative colitis. *Lancet*.

[7] Yang Q., Wang Y., Jia A., Wang Y., Bi Y., Liu G. (2021). The crosstalk between gut bacteria and host immunity in intestinal inflammation. *Journal of Cellular Physiology*.

[8] Montalban-Arques A., Chaparro M., Gisbert J. P., Bernardo D. (2018). The innate immune system in the gastrointestinal tract: role of intraepithelial lymphocytes and lamina propria innate lymphoid cells in intestinal inflammation. *Inflammatory Bowel Diseases*.

[9] Dang P. M., Rolas L., El-Benna J. (2020). The dual role of reactive oxygen species-generating nicotinamide adenine dinucleotide phosphate oxidases in gastrointestinal inflammation and therapeutic perspectives. *Antioxidants & Redox Signaling*.

[10] Choi J. H., Chung K. S., Jin B. R. (2017). Anti-inflammatory effects of an ethanol extract of Aster glehni via inhibition of NF-*κ*B activation in mice with DSS-induced colitis. *Food & Function*.

[11] Ahmed S. M. U., Luo L., Namani A., Wang X. J., Tang X. (2017). Nrf2 signaling pathway: pivotal roles in inflammation. *Biochimica et Biophysica Acta - Molecular Basis of Disease*.

[12] Jia L., Xue K., Liu J. (2020). Anticolitic effect of berberine in rat experimental model: impact of PGE2/p38 MAPK pathways. *Mediators of Inflammation*.

[13] Pais P., Almeida V., Yılmaz M., Teixeira M. C. (2020). Saccharomyces boulardii: what makes it tick as successful probiotic?. *Journal of Fungi*.

[14] Terciolo C., Dobric A., Ouaissi M. (2017). Saccharomyces boulardii CNCM I-745 restores intestinal barrier integrity by regulation of E-cadherin recycling. *Journal of Crohn's & Colitis*.

[15] Zanello G., Meurens F., Berri M., Salmon H. (2009). Saccharomyces boulardii effects on gastrointestinal diseases. *Current Issues in Molecular Biology*.

[16] Everard A., Matamoros S., Geurts L., Delzenne N. M., Cani P. D. (2014). Saccharomyces boulardii administration changes gut microbiota and reduces hepatic steatosis, low-grade inflammation, and fat mass in obese and type 2 diabetic db/db mice. *mBio*.

[17] Sougioultzis S., Simeonidis S., Bhaskar K. R. (2006). Saccharomyces boulardii produces a soluble anti-inflammatory factor that inhibits NF-kappaB-mediated IL-8 gene expression. *Biochemical and Biophysical Research Communications*.

[18] Buccigrossi V., Laudiero G., Russo C. (2014). Chloride secretion induced by rotavirus is oxidative stress-dependent and inhibited by Saccharomyces boulardii in human enterocytes. *PLoS One*.

[19] Cooper H. S., Murthy S. N., Shah R. S., Sedergran D. J. (1993). Clinicopathologic study of dextran sulfate sodium experimental murine colitis. *Laboratory Investigation*.

[20] Xiao B., Zhang Z., Viennois E. (2016). Combination therapy for ulcerative colitis: orally targeted nanoparticles prevent mucosal damage and relieve inflammation. *Theranostics*.

[21] Hu Y., Chen D., Zheng P. (2019). The bidirectional interactions between resveratrol and gut microbiota: an insight into oxidative stress and inflammatory bowel disease therapy. *BioMed Research International*.

[22] Guazelli C. F. S., Fattori V., Ferraz C. R. (2021). Antioxidant and anti-inflammatory effects of hesperidin methyl chalcone in experimental ulcerative colitis. *Chemico-Biological Interactions*.

[23] Wang X., Yu N., Peng H. (2019). The profiling of bioactives in Akebia trifoliata pericarp and metabolites, bioavailability and in vivo anti-inflammatory activities in DSS-induced colitis mice. *Food & Function*.

[24] Wang K., Jin X., Li Q. (2018). Propolis from different geographic origins decreases intestinal inflammation andBacteroidesspp. populations in a model of DSS-induced colitis. *Molecular Nutrition & Food Research*.

[25] Rodríguez-Nogales A., Algieri F., Garrido-Mesa J. (2017). Differential intestinal anti-inflammatory effects ofLactobacillus fermentumandLactobacillus salivariusin DSS mouse colitis: impact on microRNAs expression and microbiota composition. *Molecular Nutrition & Food Research*.

[26] Farombi E. O., Adedara I. A., Awoyemi O. V. (2016). Dietary protocatechuic acid ameliorates dextran sulphate sodium-induced ulcerative colitis and hepatotoxicity in rats. *Food & Function*.

[27] Tatiya-Aphiradee N., Chatuphonprasert W., Jarukamjorn K. (2018). Immune response and inflammatory pathway of ulcerative colitis. *Journal of Basic and Clinical Physiology and Pharmacology*.

[28] Cui L., Guan X., Ding W. (2021). Scutellaria baicalensis Georgi polysaccharide ameliorates DSS-induced ulcerative colitis by improving intestinal barrier function and modulating gut microbiota. *International Journal of Biological Macromolecules*.

[29] Qu S., Shen Y., Wang M., Wang X., Yang Y. (2019). Suppression of miR-21 and miR-155 of macrophage by cinnamaldehyde ameliorates ulcerative colitis. *International Immunopharmacology*.

[30] Szkaradkiewicz A., Marciniak R., Chudzicka-Strugała I. (2009). Proinflammatory cytokines and IL-10 in inflammatory bowel disease and colorectal cancer patients. *Archivum Immunologiae et Therapiae Experimentalis (Warsz)*.

[31] Peng Y., Yan Y., Wan P. (2019). Gut microbiota modulation and anti-inflammatory properties of anthocyanins from the fruits of Lycium ruthenicum Murray in dextran sodium sulfate-induced colitis in mice. *Free Radical Biology & Medicine*.

[32] Luissint A. C., Parkos C. A., Nusrat A. (2016). Inflammation and the intestinal barrier: leukocyte-epithelial cell interactions, cell junction remodeling, and mucosal repair. *Gastroenterology*.

[33] Chami B., Martin N. J. J., Dennis J. M., Witting P. K. (2018). Myeloperoxidase in the inflamed colon: a novel target for treating inflammatory bowel disease. *Archives of Biochemistry and Biophysics*.

[34] Bagaev A. V., Garaeva A. Y., Lebedeva E. S., Pichugin A. V., Ataullakhanov R. I., Ataullakhanov F. I. (2019). Elevated pre-activation basal level of nuclear NF-kappaB in native macrophages accelerates LPS-induced translocation of cytosolic NF-kappaB into the cell nucleus. *Scientific Reports*.

[35] Li B., Alli R., Vogel P., Geiger T. L. (2014). IL-10 modulates DSS-induced colitis through a macrophage-ROS-NO axis. *Mucosal Immunology*.

[36] Samoila I., Dinescu S., Costache M. (2020). Interplay between cellular and molecular mechanisms underlying inflammatory bowel diseases development-a focus on ulcerative colitis. *Cell*.

[37] Liu D., Huo X., Gao L., Zhang J., Ni H., Cao L. (2018). NF-*κ*B and Nrf2 pathways contribute to the protective effect of Licochalcone A on dextran sulphate sodium-induced ulcerative colitis in mice. *Biomedicine & Pharmacotherapy*.

[38] Khodir A. E., Said E., Atif H., ElKashef H. A., Salem H. A. (2019). Targeting Nrf2/HO-1 signaling by crocin: role in attenuation of AA-induced ulcerative colitis in rats. *Biomedicine & Pharmacotherapy*.

[39] Almeer R. S., Mahmoud S. M., Amin H. K., Abdel Moneim A. E. (2018). Ziziphus spina-christi fruit extract suppresses oxidative stress and p38 MAPK expression in ulcerative colitis in rats via induction of Nrf2 and HO-1 expression. *Food and Chemical Toxicology*.

[40] Kourakis S., Timpani C. A., de Haan J. B., Gueven N., Fischer D., Rybalka E. (2021). Targeting Nrf2 for the treatment of Duchenne muscular dystrophy. *Redox Biology*.

