# Supplementary material for: Saccharomyces boulardii Ameliorates Dextran Sulfate Sodium-Induced Ulcerative Colitis in Mice by Regulating NF-κB and Nrf2 Signaling Pathways
Source: Oxid Med Cell Longev. 2021 Jul 28;2021:1622375. doi: 10.1155/2021/1622375 (PMC8342159; doi:10.1155/2021/1622375)
Supplement: Supplementary Materials — Tables S1-S2 were listed in the Supplementary Material. [file 1622375.f1.docx]

**Table S1** Evaluation of the DAI

| Score | Weight loss (%) | Stool consistency | Occult/gross bleeding |
| --- | --- | --- | --- |
| 0 | 0 | Normal | Normal |
| 1 | 1-5 |  |  |
| 2 | 6-10 | Loose stool | Hemoccult positive |
| 3 | 11-15 |  |  |
| 4 | >15 | Diarrhea | Gross bleeding |

**Table S2** Evaluation of pathological score

| Score | Epithelial cells | Inflammatory cell infiltration |
| --- | --- | --- |
| 0 | Normal form | No infiltration |
| 1 | Goblet cell loss | Infiltration in basal layer of crypt |
| 2 | Crypt cells loss | Infiltration reaches the mucosal muscle layer |
| 3 | Large area loss of crypt cells | Infiltration to the submucosa |
